# Use of low-level laser therapy as an adjuvant therapy for phlebostatic lesions

**DOI:** 10.1590/1677-5449.202301592

**Published:** 2024-07-08

**Authors:** Lucas Dalvi Armond Rezende, Davi de Souza Catabriga, Alicia de Oliveira Pacheco, Aline de Oliveira Ramalho, Paula de Souza Silva Freitas

**Affiliations:** 1 Universidade Federal do Rio de Janeiro – UFRJ , Faculdade de Medicina, Rio de Janeiro, RJ, Brasil.; 2 Escola Superior de Ciências da Santa Casa de Misericórdia de Vitória – EMESCAM, Vitória, ES, Brasil.; 3 Universidade de São Paulo – USP, Hospital das Clínicas, São Paulo, SP, Brasil.; 4 Hospital Sírio Libanês – HSL, São Paulo, SP, Brasil.; 5 Universidade Federal do Espírito Santo – UFES, Departamento de Enfermagem, Vitória, ES, Brasil.

**Keywords:** venous insufficiency, terapia com luz de baixa intensidade, venous ulcer

## Abstract

Low-intensity laser therapy (LILT) is commonly used as an adjuvant therapy for treating injuries. This integrative literature review was carried out in the MEDLINE, LILACS, CUMED, BDENF, SPORTDiscus, Dentistry & Oral Sciences Source, Academic Source and CINAHL databases. Among the inclusion criteria were: range from 2011 to 2021, in English, Portuguese and Spanish and any study, with the exception of preprints and books. The question was answered: “What is the effectiveness described in the literature of using low-intensity laser therapy (LILT) in the treatment of venous lesions?” The wavelength used in studies varied from 635 nm of red ray to 780 nm of infrared ray, generating healing improvement at any length. LBI presented itself as a low-cost and easy-to-apply adjuvant option, alleviating pain complaints and improving healing in patients with vasculogenic lesions.

## INTRODUCTION

**Figure 1:**
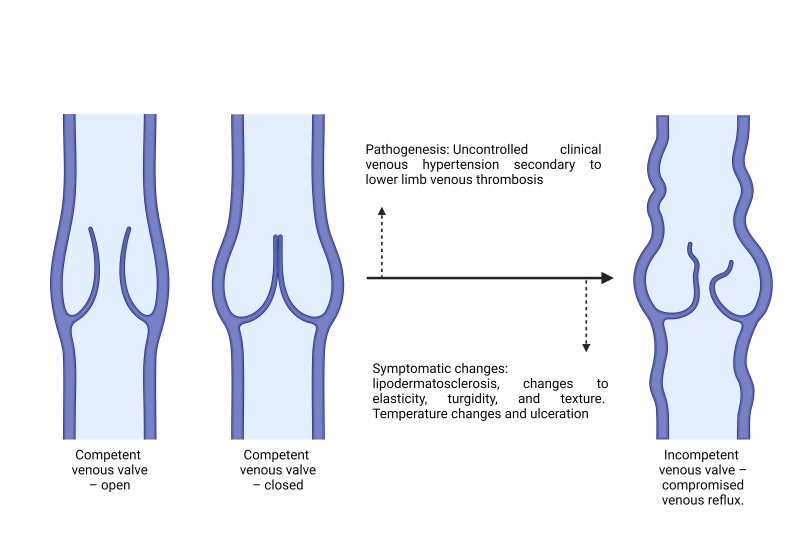
Simplified illustration of the pathophysiologic process of chronic venous insufficiency. Source: the authors (2024). Figure drawn using Biorender.

Chronic venous disease is a common disease of the circulatory system and is a significant medical problem for affected patients that imposes a considerable cost burden on the health care system. The condition encompasses a cascade of pathophysiologic consequences, the majority of which are the result of venous hypertension in the lower extremities, which in turn can have a variety of etiologies. It is common for venous hypertension to be associated with valve disorders, culminating in accumulation of blood, hypoxia, inflammation, and cell death. Figure 1 briefly summarizes its pathogenesis.^1^

Clinical signs of valve incompetence include telangiectasias, varicose veins, edemas, and skin changes such as hyperpigmentation and stiffening of the integument. In more advanced cases, without correct and early treatment, patients can develop cutaneous ulcerations, including those that are irregularly shaped and superficial, located in the distal lower limbs, at the medial malleolus, with purple-colored and hyperpigmented perilesional skin with eczema, blisters, and itching.^1,2^

The elevated pressures within the dysfunctional system are transmitted to the capillaries and veins that supply the tegumentary tissues, increasing permeability, leakage, and deposition of hemosiderin in tissues, causing changes to texture and elasticity and provoking the signs described above and ulceration itself.^3,4^

Treatment of venous ulcers involves surgical correction of the venous insufficiency, when indicated, combined with use of phlebotonic substances, such as natural and synthetic flavonoids (diosmin, for example), and hemorheologic substances, such as pentoxifylline.^2^ Other important therapies that are part of the treatment regimen for these ulcers include active compression and use of pharmacological dressings for lesion debridement.^2,5-7^ Additionally, there are now also adjuvant treatments on the market, such as low-level laser therapy (LILT), which has been used in a wide range of situations to improve patients’ clinical status.^8^

Against this background, the objective of the present article is to discuss the evidence available in the literature for use of LILT in treatment of venous ulcers, seeking options to increase the efficacy of conventional treatments for phlebostatic lesions.

## MATERIALS AND METHODS

**Figure 2:**
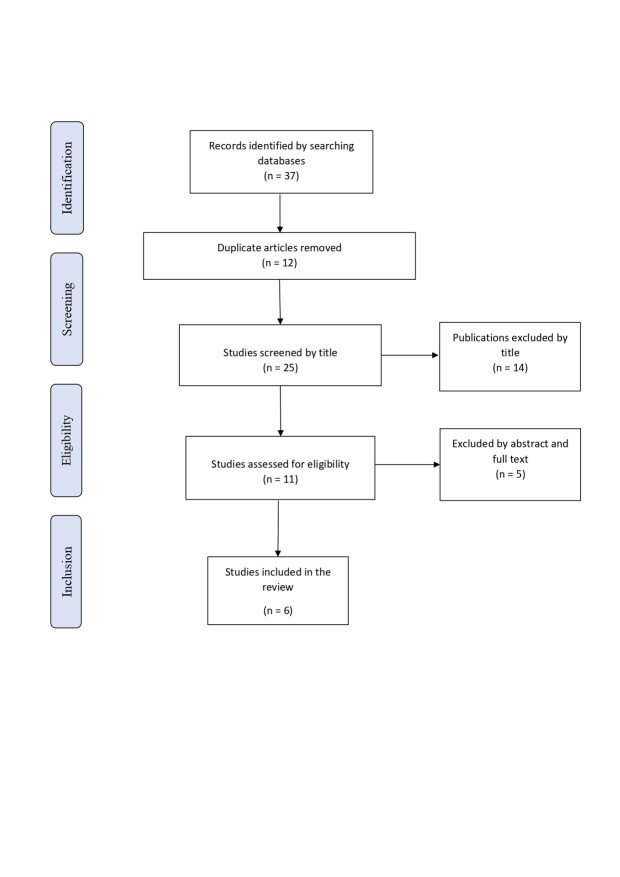
Article selection process. Source: the authors (2024).

This is an integrative literature review following recommendations set out by Botelho et al., who suggest a six-stage process, as follows: definition of a guiding question, elaboration of the topic to be studied, synthesis of inclusion and exclusion criteria, identification of studies, summary and synthesis of the review, and interpretation of the results.^9,10^Figure 2 illustrates the stages of article selection as proposed in the Preferred Reporting Items for Systematic Reviews and Meta-Analyses (PRISMA) statement.^11^

### Literature search and study selection

Studies published in English, Portuguese, and Spanish were selected from the results of searches of the databases Medical Literature Analysis and Retrieval System Online (MEDLINE), Literatura Latino Americana e do Caribe de Informação em Ciências da Saúde (LILACS), Centro Nacional de Información de Ciencias Médicas de Cuba (CUMED), Base de Dados de Enfermagem (BDENF), SPORTDiscus with Full Text, Dentistry & Oral Sciences Source, Fonte Acadêmica, and Cumulative Index to Nursing & Allied Health Literature (CINAHL). The search strategy was designed and executed by two experienced authors, independently. The following health sciences descriptors (DeCS) and medical subject headings (MeSH) were used: Úlcera varicosa” (“Venous ulcer”), “Terapia a laser” (“Laser therapy”), “Terapia com luz de baixa intensidade” (“Low-level laser therapy”), and “LILT” (“LILT”), separated by Boolean operators. The search string used was as follows: “Venous ulcer” AND “Laser therapy” AND “Low-level laser therapy” AND “LILT”, and the equivalent in Portuguese.

### Results of interest

In order to accomplish the stages described above, the study identified publications on LILT for treatment of venous ulcers to answer the following guiding question: “What is the efficacy reported in the literature for low-level laser therapy (LILT) used for treatment of venous lesions?”, which was constructed using the PICO strategy, based on the mnemonic: P – Patient, I – Intervention, C – Comparison, and O – Outcome, as summarized in Table 1.^12^

**Table 1:** Process of development of the guiding question.

P – Population	Patients with venous ulcer
I - Intervention	Use of low-level laser therapy
C- Comparison	Healthy patients
O- Outcome	Efficacy/healing

**SOURCE:** The authors (2024).

### Inclusion and exclusion criteria

The review included publications in English, Spanish, and Portuguese from the last 10 years (2011 to 2021) describing all types of study except for preprints, books, and publications that did not deal with the subject of the review, regardless of whether the full text was available. Additionally, articles were only included in the review if they discussed ulcers exclusively of venous origin and their adjuvant therapy with LILT. Articles were included if they reported use of LILT in conjunction with other types of treatment, such as photodynamic therapy and cellulose membranes.

Publications were excluded if they did not contribute to answering the guiding question; publications that were classified as books or documents; and publications that did not cover the central topic of the review.

### Data extraction

After reading and selecting the articles, they were summarized in a Microsoft Word 365 document listing year of publication, authors’ names, title of publication, laser wavelength, publication language, and a brief summary of the article content (stage 4 of the review process). This was used to construct a table summarizing the findings. Additionally, the quality of the journals of publication was also listed, with their impact factors, and also the professional qualifications of the authors, and the evidence levels of the studies.

In order to minimize possible errors or biases, study selection was performed by two independent reviewers in two steps. The first step comprised reading the titles and abstracts and the second step comprised reading full texts. In cases of disagreement, inclusion of the publications was discussed with a third reviewer with experience in the literature review method who either approved or excluded the article.

## RESULTS

The findings were summarized in Table 2. A total of six publications were included in the review. Publication years were as follows: 2012 (n = 1/16.7%), 2017 (n = 1/16.7%), 2018 (n = 3/50%), and 2020 (n = 1/16.7%). By publication language, English (n = 4/66.7%) predominated, followed by Spanish (n = 1/16.7%) and Portuguese (n = 1/16.7%).

**Table 2:** Summary of the publications selected.

Title / Identification	Year	Language	Authors	Brief conclusions
Photonic technology for the treatments of venous and arterial ulcers: Case report/A	2018	English	Carbinatto et al.^13^	The combined effects of photodynamic therapy, LILT, and cellulose biomembrane were investigated for healing of venous ulcers and the results showed a large reduction in ulcer area.
Comportamiento de las úlceras venosas de los miembros inferiores tratadas con láser de baja potencia/B	2012	Spanish	de la Cruz et al.^14^	Among the patients treated with laser, there were improvements in subjective symptoms and clinical manifestations, some ulcers healed without needing a second treatment cycle.
A Double-Blind, Placebo-Controlled Randomized Evaluation of the Effect of Low-Level Laser Therapy on Venous Leg Ulcers/C	2017	English	Vitse et al.^15^	Use of LILT can be considered a coadjuvant treatment for venous ulcers, helping with complaints of pain.
Fotobiomodulação no processo cicatricial de lesões - estudo de caso/D	2020	Portuguese	Lucio and Paula^16^	The therapy showed a positive result with marked improvement of healing and the patient’s quality of life.
Low-level laser therapy for treatment of venous ulcers evaluated with the Nursing Outcome Classification: study protocol for a randomized controlled trial/E	2018	English	Bavaresco et al.^17^	Improved quality of life and velocity and efficacy of treatment for venous ulcers.
Randomized controlled trial for treatment of pain and assessment of wound healing in chronic venous leg ulcers using near infrared laser therapy/F	2018	English	Jennings and Suggs^18^	Clinical trial still ongoing, but showing benefits for patient quality of life.

LILT = low-level laser therapy. **Source:** the authors (2024).

Based on their methodology, the articles analyzed had low or moderate evidence levels (EL), as follows: prospective randomized studies (n = 3/50%/EL = 2C), a controlled and randomized study (n = 1/16.7%/EL = 1B), and case reports (n = 2/33.3%/EL = 5).^19^ We used the Oxford evidence level classification.

Our review also analyzed the wavelength used in each study, which were in the range of 635 nm, red light, to 780 nm, in the infrared band. None of the articles selected had a dichotomous comparison of wavelengths and their benefits.

Next, in order to assess the quality of the published studies, a simple search was performed to identify the impact factor of each periodical, which is a bibliometric method for evaluating the importance of a scientific journal within its field. The impact factors observed were as follows: *Photodiagnosis Photodyn Ther* (2.894), *Rev. Cub Ang Cir Vasc* (impact factor not found), *Int J Low Extrem Wounds* (1.380), *CuidArte Enfermagem* (impact factor not found), *Trials* (1.883) and *Clinical Trials* (2.462), as listed systematically in Table 3.

**Table 3:** Relationships between publications and data.

Study	Periodical and impact factor	Qualifications of lead author	Wavelength	Study design/Oxford EL
A	*Photodiagnosis Photodyn Ther*/2.894	Pharmaceutical – Masters and Doctorate in Pharmaceutical Sciences	660 nm (red)	Case report/EL 5
B	*Rev Cub Ang Cir Vasc*/impact factor not found	Qualifications not found	610 nm (red) – 780 nm (infrared)	Prospective randomized study/EL 2c
C	*Int J Low Extrem Wounds*/1.380	Physician – plastic surgeon	635 nm	Prospective randomized study/EL 2c
D	*CuidArte Enferm*/impact factor not found	Nurse – Masters in Nursing	Wavelength not reported	Case report/EL 5
E	*Trials*/1.883	Nurse – Masters and Doctorate in Nursing	660 nm (red)	Prospective randomized study/EL 2c
F	*Clinical Trials*/2.462	Qualifications not found	Wavelength not reported	Randomized, controlled clinical trial/EL 1b

EL = evidence level. **Source:** the authors (2024).

Additionally, a search was conducted to identify the professional qualifications of the lead authors of the studies, which identified a predominance of Nursing bachelor’s degrees (n = 2/33.3%), followed by a bachelor’s degree in Pharmacy (n = 1/16.7%) and a bachelor’s degree in Medicine (n = 1/16.7%). It was not possible to identify the professional qualifications of the lead authors of two of the studies.

Analysis of the results of the primary outcomes of the studies showed that all of the articles (N = 6/100%) reported that patients responded well, with improvements in the healing process and long-term pain and improved quality of life because of the rapid healing and the reduced time taken to heal, reducing chronification.

## DISCUSSION

Analysis of the results of the studies revealed evidence of a positive effect of LILT during all of the phases of healing and treatment of vascular ulcers, promoting better healing of wounds of vascular origin.

Wound healing is a multifactorial event involving several events related to wound etiology and to influential local and/or systemic factors. Didactically, the healing process is divided into three phases: inflammation, proliferation, and maturation, which implies events such as coagulation, recruitment of inflammatory cells, and synthesis of a provisional matrix at the site of the injury.^20^

Photobiomodulation with LILT modifies several biological effects, especially those involved in cell proliferation during healing. This phase requires a constant equilibrium between apoptosis of existing cells and synthesis of new cells. Certain situations are critical during this phase and any error or change affecting the healing process can allow the lesion to become chronic.^20^

**Figure 3:**
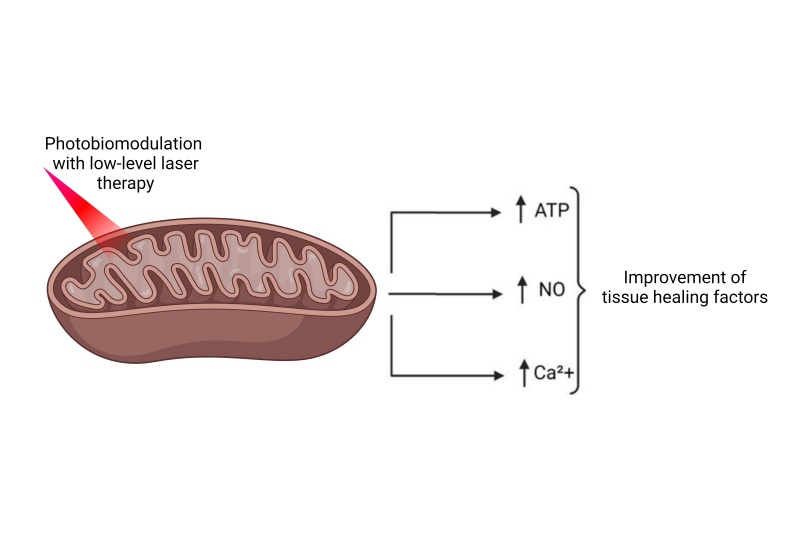
Mechanism of mitochondrial action with low-level laser therapy stimulation. Source: the authors (2024). Figure drawn using Biorender.

It is known that mitochondria are the principal cellular photoreceptors of the photons emitted during the photobiomodulation process, which are absorbed by mitochondrial chromophores in the skin, promoting a twofold increase in activity of the mitochondrial respiratory chain. As illustrated in Figure 3, this process results in elevated ATP levels in superficial and central nervous tissues and also provokes release of nitric oxide (NO), reactive oxygen species (ROS), and intracellular calcium. It is known that these factors provoke improved wound healing and avert tissue necrosis in healthy rats with no predisposing genetic comorbidities.^21,22^

Fornaguera et al. published a prospective descriptive study of 60 patients with confirmed medical diagnosis of lower limb venous insufficiency and severe venous ulcers. Their sample comprised 41 women and 19 men aged 20 to 89 years, none of whom had systemic complications.^14^ The study employed a laser system with a continuous emission semiconductor diode to administer 15 LILT sessions over 3 weeks to each patient, using doses of 650 nm red laser and 780 nm infrared laser to achieve analgesic and anti-inflammatory effects, promoting tissue regeneration. These cases had successful outcomes using laser to treat phlebostatic lesions and the authors observed that 67% (n=40) of the patients achieved total epithelialization after 3 weeks of treatment.^14^

In line with that result was a French, prospective, randomized, double-blind study that selected 24 participants aged 40 years or older with a 6-week history of non-healing venous ulcers of the lower limbs. These participants received two 20-minute treatments per week until complete wound healing was observed at 2 weeks or up to 12 consecutive weeks of administration of the treatment. After 12 weeks of treatment, 23% of the test group (n = 13) and 18% of the placebo group (n = 11) achieved complete wound healing and there was a potential reduction in patients’ complaints of pain, with up to 85% reduction in pain among patients with lesions.^15^ LILT promotes increased angiogenesis by increasing expression of hypoxia inducible factor alpha (HIF-α) and vascular endothelial growth factor (VEGF) while also reducing activity of cellular matrix metalloproteinase-2 (MMP-2) in vitro,^23^ and possible improvement of complaints of pain related to venous ulcers.^18^

A randomized clinical trial conducted in Brazil by Bavaresco et al.^17^ followed 40 patients in nursing consultations with the objective of comparing conventional venous ulcer treatment only versus treatment with LILT for 16 weeks. Their results were in line with reports describing improvement in the healing process, reducing the time taken for tissue regeneration. In the intervention group, the first venous ulcer healed during the second week of treatment, whereas healing only occurred in the seventh week in the control group. The study reported a statistically significant difference with *p*=0.031. The results of this study contributed to advances in treatment of ulcers and the clinical practice of specialist nurses and physicians in the field.^24^

Lúcio and Paula published case report of a white male patient with a history of diabetes mellitus, systemic arterial hypertension, smoking and left saphenectomy with a venous ulcer of the left limb. At a first consultation, the lesion was free from edema, with bilateral dermatosclerosis and wet necrosis, with moderate exudate. He was treated with seven sessions of LILT with a low power 100mW diode laser, with intralesional administration of 2 joules of red and infrared light. After 55 days of treatment with LILT in conjunction with high technology dressings, the authors observed considerable improvement in the necrotized tissue, reduced exudate, and total retraction of the ulcer, with complete healing.^16^

In line with the study described above, Carbinatto et al. reported the case of a 50-year-old Caucasian man with bilateral lower limb lesions, of venous origin in the left lower limb and arterial etiology on the right, with onset 10 years previously. This patient was treated with three techniques in conjunction (LILT, cellulose membrane, and photodynamic therapy with blue methylene) twice per week for 3 months. The lesioned areas were irradiated for 12 minutes with 450 nm light at an intensity of 75 mW/cm^2^, with a dosage of 54 J/cm^2^. After 2 days, a treatment protocol with LILT was initiated with 660 nm red laser light with a punctual and continuous technique, twice per week, for 30 seconds, at 10 J/cm^2^. It was concluded that the synergic effect of these therapies enabled the healing process to be accelerated and reduced the size of the ulcers, achieving an 85% reduction in lesion area.^13^

## CONCLUSIONS

It was found, according to the summary of these studies, that LILT was able to promote beneficial effects for treatment of vascular ulcers. The studies highlighted its role in epithelialization and stimulation of healing factors, such as VEGF, reduction of proinflammatory factors, and release of nitric oxide. It is believed that LILT is an important treatment option, with tissue regeneration capabilities that favor its use.

It is believed that LILT can provoke acceleration of the healing process, resulting in reduced use of dressings and other high-cost products, thereby economizing expenditure for the health system and promoting increased quality of life for individuals suffering with chronic venous lesions.

One limitation of our review is the number of studies published in the subject with small numbers of participants and another is the integrative nature of the review. It is important to conduct studies with robust methodology and detailed and standardized clinical protocols so that LILT can be incorporated into the Brazilian public health care system, given its low cost, ease of application, and absence of side effects.
