# Supplementary material for: Use of low-level laser therapy as an adjuvant therapy for phlebostatic lesions
Source: J Vasc Bras. 2024 Jul 8;23:e20230159. doi: 10.1590/1677-5449.202301592 (PMC11296685; doi:10.1590/1677-5449.202301592)
Supplement: Translation

# Translation: Uso de laserterapia de baixa intensidade em lesões flebostáticas como terapia adjuvante

**DOI:** 10.1590/1677-5449.202301591

**Authors:** Lucas Dalvi Armond Rezende, Davi de Souza Catabriga, Alicia de Oliveira Pacheco, Aline de Oliveira Ramalho, Paula de Souza Silva Freitas

**Affiliations:** 1 Universidade Federal do Rio de Janeiro – UFRJ , Faculdade de Medicina, Rio de Janeiro, RJ, Brasil.; 2 Escola Superior de Ciências da Santa Casa de Misericórdia de Vitória – EMESCAM, Vitória, ES, Brasil.; 3 Universidade de São Paulo – USP, Hospital das Clínicas, São Paulo, SP, Brasil.; 4 Hospital Sírio Libanês – HSL, São Paulo, SP, Brasil.; 5 Universidade Federal do Espírito Santo – UFES, Departamento de Enfermagem, Vitória, ES, Brasil.

**Keywords:** insuficiência venosa, terapia com luz de baixa intensidade, úlcera varicosa

## Abstract

A laserterapia de baixa intensidade (LBI) é comumente utilizada como terapia adjuvante para tratamento de lesões. Essa revisão integrativa de literatura foi realizada nas bases MEDLINE, LILACS, CUMED, BDENF, *SPORTDiscus, Dentistry & Oral Sciences Source, Fonte Acadêmica* e CINAHL. Entre os critérios de inclusão estavam: intervalo de 2011 a 2021, nos idiomas inglês, português e espanhol e qualquer estudo, com exceção de *preprints* e livros. Respondeu-se à questão: “Qual a eficácia descrita na literatura do uso da laserterapia de baixa intensidade (LBI) no tratamento de lesões venosas?”. O comprimento de onda usado nos estudos variou de 635 nm de raio vermelho ah 780 nm de raio infravermelho, gerando melhora cicatricial em qualquer comprimento. A LBI apresentou-se como uma opção coadjuvante de baixo custo e de fácil aplicação, amenizando queixas álgicas e melhorando a cicatrização de pacientes com lesões vasculogênicas.

## INTRODUÇÃO

A doença venosa crônica é uma doença comum do sistema circulatório, sendo representada como um problema médico significativo para os pacientes, elevando substancialmente os gastos do sistema de saúde. Tal condição engloba uma cascata de consequências fisiopatológicas decorrentes majoritariamente da hipertensão venosa nas extremidades de membros inferiores, que pode ter variadas etiologias. É comum que a hipertensão venosa esteja associada a alteração valvar, culminando no acúmulo de sangue, hipóxia, inflamação e morte celular. Sua patogênese é representada brevemente na Figura 1^1^ .

**Figura 1:** Processo fisiopatológico simplificado da insuficiência venosa crônica. Fonte: Autoral (2021). Figura sintetizada pelo aplicativo Biorender. Legenda: Insuficiência venosa crônica – IVC.

Os sinais clínicos que acompanham a incompetência valvar incluem telangiectasias, varizes, edemas e alterações cutâneas como hiperpigmentação e endurecimento tegumentar. Já em casos mais avançados, sem o tratamento precoce e correto, os pacientes podem desenvolver ulcerações cutâneas, entre as quais encontram-se aquelas de formato irregular e superficial, localização na porção distal dos membros inferiores no maléolo medial, com pele perilesional de coloração purpúrica e hiperpigmentada com eczema, vesícula e prurido^1,2^ .

Elevadas pressões nesse sistema defeituoso são transmitidas aos capilares e veias que irrigam o tecido tegumentar, promovendo um aumento da permeabilidade, extravasamento e deposição de hemossiderina no tecido, o que causa alterações de textura e elasticidade, provocando os sinais descritos acima e a ulceração^3,4^ .

O tratamento das úlceras venosas envolve a correção cirúrgica da insuficiência venosa, quando indicada, associada ao uso de substâncias flebotônicas como flavonoides naturais e sintéticos (por exemplo, a diosmina), e substâncias hemorreológicos, como a pentoxifilina^2^ . Outras terapias se destacam para o esquema de tratamento dessas úlceras, como a realização de compressão ativa e o uso de coberturas farmacológicas para o debridamento da lesão^2,5-7^ . Outrossim, o mercado atual apresentou tratamentos adjuvantes, como o uso da laserterapia de baixa intensidade (LBI), a qual tem sido usada em diversas situações para melhora do estado clínico do paciente^8^ .

Nesse contexto, o presente artigo objetiva discutir sobre as evidências disponíveis na literatura com relação ao uso de LBI no tratamento de lesões venosas, buscando alternativas que potencializem os tratamentos convencionais das lesões flebostáticas.

## MATERIAIS E MÉTODOS

Trata-se de uma revisão integrativa de literatura que segue as recomendações dos autores Botelho et al., as quais sugerem a sua realização em seis etapas diferentes, a saber: elaboração de uma questão norteadora, elaboração da temática estudada, síntese dos critérios de inclusão e exclusão, identificação dos estudos, sumarização e síntese da revisão com a interpretação dos resultados^9,10^ . Foram representadas as etapas de seleção de artigos propostas pela declaração de Itens de Relatório Preferenciais para Revisão Sistemática e Meta-análises (PRISMA), conforme apresentado na Figura 2^11^ .

**Figura 2:** Processo de seleção dos artigos. Fonte: Autoral (2024).

### Pesquisa de literatura e seleção dos estudos

Foram selecionados estudos nos idiomas inglês, português e espanhol, a partir de buscas nas bases de dados *Medical Literature Analysis and Retrieval System Online* (MEDLINE), Literatura Latino Americana e do Caribe de Informação em Ciências da Saúde (LILACS), Centro nacional de Informações de Ciências Médicas de Cuba (CUMED), Base de Dados de Enfermagem (BDENF)**,** *SPORTDiscus with Full Text*, *Dentistry & Oral Sciences Source, Fonte Acadêmica* e *Cumulative Index to Nursing & Allied Health Literature* (CINAHL)**.** A estratégia de busca foi desenhada e conduzida por dois autores experientes, de modo independente. Foram utilizados os seguintes descritores de ciência em saúde (DeCS) e medical subject headings (MeSH): “Úlcera varicosa” (“Venous ulcer”), “Terapia a laser” (“Laser therapy”) e “Terapia com luz de baixa intensidade” (“Low-level laser therapy”) e “LLLT” (“LLLT”), os quais foram separados por meio dos operadores booleanos. A pesquisa resumiu-se em: “Úlcera varicosa” AND “Terapia a laser” AND “Terapia com luz de baixa intensidade” AND “LLLT”, sendo traduzida corretamente para o idioma inglês.

### Resultados de interesse

Para seguir os passos descritos, este estudo buscou identificar as produções sobre LBI no tratamento de lesões venosas através da seguinte questão norteadora: “Qual a eficácia descrita na literatura do uso da Laserterapia de Baixa Intensidade (LBI) no tratamento de lesões venosas?”, a qual foi montada por meio da estratégia PICO, em que se faz presente o mnemônico: P – Paciente, I – Intervenção, C- Comparação e O – Desfecho/*Outcome*, e esquematizada na Tabela 1^12^ .

**Tabela 1:** Processo de síntese da questão norteadora.

P – População	Pacientes com úlcera venosa
I - Intervenção	Uso de laserterapia de baixa intensidade
C- Comparação	Pacientes hígidos
O- Desfecho	Eficácia/cicatrização

**FONTE:** Autoral (2024).

### Critérios de inclusão e exclusão

Foram incluídas publicações dos últimos 10 anos (2011 a 2021), sendo nos idiomas inglês, espanhol e português, todos os tipos de estudo, com exceção de *preprints*, livros e produções que não abarcaram a temática do artigo, estando o texto completo disponível ou não. Além disso, os artigos incluídos na revisão abarcam exclusivamente úlceras de origem venosa, bem como sua terapêutica adjuvante voltada para o uso de LBI. Foram incluídos artigos que relatam o uso da LBI associada com outras formas de tratamento, como terapia fotodinâmica e uso de membrana de celulose.

Foram excluídas as produções que não contemplavam a questão norteadora descrita; além disso, foram desconsiderados para esta revisão artigos que tiveram como classificação livros e documentos, bem como aqueles não abarcaram a temática central da pesquisa.

### Extração de dados

Após a leitura e seleção dos artigos, estes foram sumarizados em um documento do Microsoft Word Office *365* contendo ano de publicação, nome dos autores, título da publicação, comprimento de onda, idioma e breve resumo sobre o conteúdo do artigo, procedimento determinado como o passo número 4. Por meio dele, sintetizou-se uma tabela com os achados. Outrossim, analisou-se a qualidade das revistas de publicação, com o fator de impacto, bem como a formação profissional dos autores e o nível de evidência do estudo.

Com o intuito de minimizar prováveis erros ou vieses, a seleção foi realizada por dois revisores independentes e organizada em duas etapas. Na primeira etapa, realizou-se a leitura do título e do resumo e, na segunda, fez-se a leitura do texto completo. Nos casos em que ocorreram desacordos, foi solicitada a opinião de um terceiro revisor experiente no método de revisões de literatura, que realizou a aprovação ou exclusão do artigo.

## RESULTADOS

Os achados foram sintetizados na Tabela 2. Incluíram-se no total seis produções. Foram encontrados os seguintes anos de publicação: 2012 (n = 1/16,7%), 2017 (n = 1/16,7%), 2018 (n = 3/50%) e 2020 (n = 1/16,7%). Ademais, de acordo com o seu idioma de origem, as produções selecionadas tiveram como idioma predominante o inglês (n = 4/66,7%), além dos idiomas espanhol (n = 1/16,7%) e português (n = 1/16,7%).

**Tabela 2:** Sumarização das produções selecionadas.

Título / Identificação	Ano	Idioma	Autor	Breve conclusão
Photonic technology for the treatments of venous and arterial ulcers: Case report/A	2018	Inglês	Carbinatto et al.^13^	Os efeitos combinados da terapia fotodinâmica, LBI e biomembrana de celulose foram investigados na cicatrização de feridas venosas, e os resultados mostram uma grande redução da área de úlcera.
Comportamiento de las úlceras venosas de los miembros inferiores tratadas con láser de baja potencia/B	2012	Espanhol	de la Cruz et al.^14^	Nos pacientes tratados com laser, notou-se melhora nos sintomas subjetivos e nas manifestações clínicas, algumas úlceras que cicatrizaram sem a necessidade de um segundo ciclo de tratamento.
A Double-Blind, Placebo-Controlled Randomized Evaluation of the Effect of Low-Level Laser Therapy on Venous Leg Ulcers/C	2017	Inglês	Vitse et al.^15^	O uso de LBI pode ser considerado um tratamento coadjuvante para úlceras venosas, auxiliando nas queixas álgicas.
Fotobiomodulação no processo cicatricial de lesões - estudo de caso/D	2020	Português	Lucio e Paula^16^	A terapia mostrou resultado positivo, com melhora acentuada da cicatrização e da qualidade de vida do paciente.
Low-level laser therapy for treatment of venous ulcers evaluated with the Nursing Outcome Classification: study protocol for a randomized controlled trial/E	2018	Inglês	Bavaresco et al.^17^	Melhorou a qualidade de vida, a velocidade e a eficácia do tratamento de lesões venosas.
Randomized controlled trial for treatment of pain and assessment of wound healing in chronic venous leg ulcers using near infrared laser therapy/F	2018	Inglês	Jennings e Suggs^18^	Ensaio clínico ainda em andamento, contudo, aponta benefícios da qualidade de vida do paciente.

LBI = laserterapia de baixa intensidade. **Fonte:** Autoral (2024).

Com base na metodologia dos artigos analisados, encontraram-se diversos estudos com baixo ou moderado nível de evidência (NE), quais sejam: estudos prospectivos randomizados (n = 3/50%/NE = 2C), ensaio controlado e randomizado (n = 1/16,7%/NE = 1B), e relatos de caso (n = 2/33,3%/NE = 5)^19^ . Consideramos o uso da classificação de Oxford para nível de evidência.

Nosso artigo também analisou o comprimento de onda utilizado por cada estudo, entre os quais identificou-se um intervalo de 635 nm de raio vermelho a 780 nm de raio infravermelho. Entre os artigos selecionados, não houve dicotomia na comparação entre os comprimentos de onda e seus benefícios.

Outrossim, com a finalidade de avaliar a qualidade dos estudos publicados, realizou-se uma busca simples sobre o fator de impacto das revistas, o qual é um método bibliométrico para avaliar a importância de periódicos científicos em suas respectivas áreas. Os fatores de impacto encontrados foram os seguintes: *Phostodiagnosis Photodyn Ther* (2.894), *Rev. Cub Ang Cir Vasc* (não identificado), *Int J Low Extrem Wounds* (1.380), *CuidArte Enfermagem* (não identificado), *Trials* (1.883) e *Clinical Trials* (2.462), conforme elucidado sistematicamente na Tabela 3.

**Tabela 3:** Relação entre produção e dados.

Estudo	Revista e fator de impacto	Formação do autor principal	Comprimento de onda	Desenho do estudo/NE de Oxford
A	*Photodiagnosis Photodyn Ther*/2.894	Farmacêutica – Mestre e Doutora em Ciências Farmacêuticas	660 nm (vermelho)	Relato de caso/NE 5
B	*Rev Cub Ang Cir Vasc*/não identificado	Não identificado	610 nm (vermelho) – 780 nm (infravermelho)	Estudo prospectivo randomizado/NE 2c
C	*Int J Low Extrem Wounds*/1.380	Médico – cirurgião plástico	635 nm	Estudo prospectivo randomizado/NE 2c
D	*CuidArte Enferm*/não identificado	Enfermeira – Mestre em Enfermagem	Não relatado	Relato de caso/NE 5
E	*Trials*/1.883	Enfermeira – Mestre e Doutora em Enfermagem	660 nm (vermelho)	Estudo prospectivo randomizado/NE 2c
F	*Clinical Trials*/2.462	Não identificado	Não relatado	Ensaio clínico controlado randomizado/NE 1b

NE = nível de evidência. **Fonte:** Autoral (2024).

Ademais, foi realizada busca acerca da formação profissional dos autores principais dos estudos, havendo predominância da formação de bacharel em Enfermagem (n = 2/33,3%), seguido de bacharel em Farmácia (n = 1/16,7%) e bacharel em Medicina (n = 1/16,7%). Não foi possível identificar a formação profissional do autor principal de dois estudos.

Quando abordado quanto aos resultados dos desfechos primários, compreendeu-se que, em todos os artigos selecionados (N = 6/100%), os pacientes responderam beneficamente quanto à melhora do processo cicatricial, melhora das queixas álgicas a longo prazo, melhora da qualidade de vida dos portadores devido à rápida cicatrização e redução no tempo de cicatrização, propiciando menor cronificação.

## DISCUSSÃO

A análise dos resultados dos estudos selecionados evidenciou um efeito positivo da ação da LBI em todas as fases de cicatrização e no tratamento de lesões vasculogênicas, promovendo uma melhora cicatricial da ferida de origem venosa.

A cicatrização de uma ferida é um evento multifatorial, que envolve diversos eventos relacionados à etiologia da lesão e a fatores influenciadores locais e/ou sistêmicos. O processo cicatricial é didaticamente dividido em três fases: inflamação, proliferação e maturação, o qual implica em eventos como a coagulação, o recrutamento de células inflamatórias e a síntese de uma matriz provisória no local da lesão^20^ .

A fotobiomodulação por meio da LBI modifica diversos efeitos biológicos, especialmente aqueles envolvidos na proliferação celular durante a cicatrização. Essa fase necessita de equilíbrio constante entre a apoptose de células existentes e a síntese de novas células. Determinadas situações são críticas nessa fase, fazendo com que qualquer erro ou alteração do processo cicatricial possa cronificar a lesão^20^ .

Sabe-se que as mitocôndrias são os principais fotorreceptores celulares de fótons emitidos durante o processo da fotobiomodulação, os quais são absorvidos pelos cromóforos mitocondriais na pele, promovendo aumento de duas vezes na atividade da cadeia respiratória mitocondrial. Tal processo, ilustrado na Figura 3, resulta em níveis aumentados de ATP nos tecidos superficiais e nervoso central, além de causar uma liberação de óxido nítrico (NO), de espécies reativas de oxigênio (ROS) e de cálcio intracelular. Sabe-se que esses fatores provocam melhora da cicatrização de feridas e evitam a necrose tecidual em ratos hígidos e sem comorbidades genéticas predispostas^21,22^ .

**Figura 3:** Mecanismo de ação mitocondrial com estimulação de laserterapia de baixa intensidade. Fonte: Autoral (2024). Figura sintetizada pelo aplicativo Biorender.

Fornaguera et al. realizaram um estudo descritivo-prospectivo em 60 pacientes com diagnóstico médico confirmado para insuficiência venosa de membros inferiores com a presença de lesão venosa grave. Sua amostra compreendeu 41 mulheres e 19 homens entre 20 e 89 anos, dos quais nenhum apresentava complicações sistêmicas^14^ .

No mesmo estudo, utilizou-se um equipamento de laser com diodo semicondutor de emissão contínua, foram aplicadas 15 sessões de LBI por 3 semanas em cada paciente, sendo dosagem de 650 nm de laser vermelho, e outro com comprimento de 780 nm de laser infravermelho, a fim de que se alcançasse uma finalidade analgésica e anti-inflamatória, promovendo a regeneração tecidual. Os casos evoluíram para sucesso na utilização do laser em lesões flebostáticas, observando que 67% (n=40) dos pacientes alcançaram uma epitelização total após 3 semanas de tratamento^14^ .

Coaduna com esse resultado um estudo francês, prospectivo, randomizado e duplo-cego, o qual selecionou 24 participantes com 40 anos ou mais com histórico de 6 semanas de úlcera venosa não cicatrizante em membros inferiores. Os participantes receberam dois tratamentos de 20 minutos por semana até que a cicatrização completa da ferida fosse avaliada em 2 semanas ou até 12 semanas consecutivas de administração do tratamento. Após 12 semanas de tratamento, 23% do grupo de teste (n = 13) e 18% do grupo de placebo (n = 11) alcançaram a cicatrização completa da ferida, e houve ainda redução potencial nas queixas álgicas dos pacientes, com melhora de até 85% nas dores dos portadores das lesões^15^ . A LBI promove o aumento da angiogênese por aumento da expressão do fator alfa induzível hipóxico (HIF-α) e do fator de crescimento endotelial vascular (VEGF), juntamente com a diminuição da atividade da metaloproteinase-2 da matriz celular (MMP-2), *in vitro* ^23^ , e possível melhora em quadros de queixa álgica relacionada à UV^18^ .

O ensaio clínico randomizado brasileiro, realizado por Bavaresco et al.,^17^ acompanhou 40 pacientes em consultas de enfermagem com objetivo de comparar o tratamento convencional isolado da UV *versus* tratamento com LBI por 16 semanas. Seus resultados coadunam com os relatos acerca da melhora do processo cicatricial, reduzindo tempo de regeneração tecidual. A cicatrização da primeira UV no grupo intervenção foi observada na segunda semana de tratamento, sendo que no grupo controle a cicatrização ocorreu apenas na sétima semana. O estudo aponta diferença estatística significativa com *p*=0,031. Os resultados deste estudo contribuem para o avanço do tratamento de feridas e prática clínica de enfermeiros e médicos especialistas na área^24^ .

Lúcio & Paula descreveram um relato de caso de um paciente do sexo masculino, branco e com histórico de diabetes *mellitus*, hipertensão arterial sistêmica, tabagismo e safenectomia de membro esquerdo com uma lesão venosa no mesmo membro. Na primeira consulta, a lesão apresentava-se sem edema, com dermatoesclerose bilateral e necrose úmida com exsudação moderada. Foi estabelecida como tratamento a LBI com laser de diodo de baixa potência de 100mW por sete sessões, aplicando-se 2 joules de luz vermelha e infravermelha intralesional. Após o 55º dia de tratamento com LBI em conjunto com coberturas de alta tecnologia, observou-se que houve importante melhora do tecido necrosado, diminuição do exsudato e retração total da lesão e completa cicatrização^16^ .

Em consonância ao estudo supracitado, Carbinatto et al. relatou o caso de um homem caucasiano de 50 anos com lesão bilateral em membro inferior, sendo o membro inferior esquerdo de origem venosa e o direito de etiologia arterial, com duração de 10 anos. A paciente foi submetida a três técnicas combinadas (LBI, aplicação de membrana de celulose e terapia fotodinâmica com azul de metileno) duas vezes por semana por 3 meses. As áreas das lesões foram irradiadas por 12 minutos com 450 nm e intensidade de 75 mW/cm^2^ com dosagem de 54 J/cm^2^. Após 2 dias, iniciou-se o protocolo de tratamento com LBI, com 660 nm de laser vermelho com técnica pontual e contínua, duas vezes por semana, por 30 segundos com 10 J/cm^2^. Concluiu-se que o efeito sinérgico das terapias, possibilitou a aceleração do processo cicatricial e redução do tamanho das lesões, tendo como redução de 85% da área lesional^13^ .

## CONCLUSÃO

Identificou-se, de acordo com a síntese dos estudos, que a LBI foi capaz de promover ações benéficas no que se refere ao tratamento de lesões vasculogênicas. Destaca-se seu papel na epitelização e no estímulo de fatores de cicatrização, como VEGF, redução de fatores pró-inflamatórios e liberação de óxido nítrico. Acredita-se que a LBI é uma importante opção terapêutica, com capacidades regenerativas teciduais favoráveis ao seu uso.

Acredita-se que o uso da LBI pode culminar em aceleração do processo de cicatrização, com utilização por menos tempo de coberturas e correlatos de alto custo, reduzindo assim os custos para o sistema de saúde e promovendo mais qualidade de vida aos indivíduos que sofrem com lesões vasculogênicas crônicas.

Nota-se que nossa revisão possui como limitação a quantidade de pesquisas publicadas sobre a temática com número limitado de participantes, bem como o tipo de revisão realizada, sendo do tipo integrativa. Salienta-se a importância de estudos com metodologia robusta e protocolos clínicos detalhados e padronizados para que a LBI se torne uma realidade incorporada no serviço público de saúde brasileiro, dado seu baixo custo, fácil manuseio e ausência de efeitos colaterais.
